# Differential retention of transposable element-derived sequences in outcrossing Arabidopsis genomes

**DOI:** 10.1186/s13100-019-0171-6

**Published:** 2019-07-17

**Authors:** Sylvain Legrand, Thibault Caron, Florian Maumus, Sol Schvartzman, Leandro Quadrana, Eléonore Durand, Sophie Gallina, Maxime Pauwels, Clément Mazoyer, Lucie Huyghe, Vincent Colot, Marc Hanikenne, Vincent Castric

**Affiliations:** 1 Univ. Lille, CNRS, UMR 8198 - Evo-Eco-Paleo, F-59000 Lille, France; 2 URGI, INRA, Université Paris-Saclay, 78026 Versailles, France; 3 InBioS - PhytoSystems, Functional Genomics and Plant Molecular Imaging, University of Liège, 4000 Liège, Belgium; 4 IBENS, Département de Biologie, Ecole Normale Supérieure, CNRS, Inserm, PSL Research University, F-75005 Paris, France

**Keywords:** Transposable elements, Arabidopsis, Genome evolution, Comparative genomics

## Abstract

**Background:**

Transposable elements (TEs) are genomic parasites with major impacts on host genome architecture and host adaptation. A proper evaluation of their evolutionary significance has been hampered by the paucity of short scale phylogenetic comparisons between closely related species. Here, we characterized the dynamics of TE accumulation at the micro-evolutionary scale by comparing two closely related plant species, *Arabidopsis lyrata* and *A. halleri*.

**Results:**

Joint genome annotation in these two outcrossing species confirmed that both contain two distinct populations of TEs with either ‘recent’ or ‘old’ insertion histories. Identification of rare segregating insertions suggests that diverse TE families contribute to the ongoing dynamics of TE accumulation in the two species. Orthologous TE fragments (i.e. those that have been maintained in both species), tend to be located closer to genes than those that are retained in one species only. Compared to non-orthologous TE insertions, those that are orthologous tend to produce fewer short interfering RNAs, are less heavily methylated when found within or adjacent to genes and these tend to have lower expression levels. These findings suggest that long-term retention of TE insertions reflects their frequent acquisition of adaptive roles and/or the deleterious effects of removing nearly neutral TE insertions when they are close to genes.

**Conclusion:**

Our results indicate a rapid evolutionary dynamics of the TE landscape in these two outcrossing species, with an important input of a diverse set of new insertions with variable propensity to resist deletion.

**Electronic supplementary material:**

The online version of this article (10.1186/s13100-019-0171-6) contains supplementary material, which is available to authorized users.

## Background

Transposable elements (TEs) are repeated elements found almost universally in eukaryotic genomes that can proliferate by high-jacking a variety of cellular processes. They are believed to be the substrate over which the non-coding fraction of the genome is formed in the long term [1] and contribute a large fraction of genome size variation across taxa, representing as much as 85% of the maize and barley genome and around 20% in *A. thaliana* [2–5]. Their spread in genomes is limited by mechanisms to suppress their transposition activity by host defense mechanisms including the production of dedicated classes of small non-coding RNAs (piRNA and siRNA) causing transcriptional silencing by RNA-dependent DNA methylation (RdDM) [6].

In spite of their quantitative importance, the evolutionary significance of TEs has been the subject of constant debate in the field. Their discovery was immediately followed by the interpretation that they must represent important “controlling elements” [7] that confer selective advantages to the organism and are a major “fuel” for evolution [6, 8]. This interpretation was soon challenged by the realization that TEs propagate in a largely selfish manner, and a large body of literature has considered them essentially as genomic parasites [9]. Over the last decade, however, molecular studies have reported convincing examples of TEs determining important evolutionary novelties and contributing to essential biological functions such as the rewiring of entire transcriptional networks [10]. Several iconic examples of rapid adaptive evolution have been linked to TE insertions such as the industrial melanism in the peppered moth [11] or the change in branching pattern that contributed to maize domestication [12]. Thanks to the regulatory elements they carry, TEs have also the capacity to confer environmental responsiveness to neighboring genes [13–15]. Hence, the duality of TEs, seen either as purely deleterious or as powerful drivers of rapid adaptive evolution has not been resolved today and the way natural selection is acting on TEs and how they accumulate in host genomes remain important questions in evolutionary genomics [16–18].

To achieve a more balanced view of TE evolution, one must therefore consider their accumulation as resulting from a complex balance between the rate and genomic locations at which they insert, the variety of their deleterious or beneficial effects and the rate at which they are removed from the genome through various recombination processes (reviewed in [19]). The landscape of TE abundance across the genome provides hints about the relative impact of these different forces. In Drosophila, recombination appears to play an important role in shaping the TE landscape, as TEs are rare in regions with a high rate of recombination and their population frequency negatively correlates with recombination [20]. In contrast, TE density does not correlate with recombination in *A. thaliana* [21], but distance to the nearest gene is strongly associated with disturbance of expression [22]. In this species, the deleterious effect of TEs thus seems to be mediated directly by their presence itself rather than indirectly by their tendency to cause ectopic recombination [21]. Hence, while examining abundance of TEs along a single genome provides insight into the selective forces involved, this correlative approach is inherently limited to a snapshot, with the caveat that a given pattern can arise from distinct evolutionary processes. For instance, the observation that TEs are typically found close to genes with low levels of expression can be due to either an insertion bias, a tendency of TE insertions to reduce the expression of adjacent genes, or generally weaker deleterious effects of TE insertions when adjacent genes are lowly rather than highly expressed [22, 23].

Different species exhibit strikingly diverse complements of TE families and superfamilies, demonstrating that evolutionary changes of this fraction of the genome can be dramatic. For instance, the majority of TEs in the pear genome [24] belong to the *Copia* superfamily, while in papaya [25] the same superfamily represents only a small fraction. It is unclear from comparing such distantly related species how fast these changes can take place, but striking differences have been observed even within species, with e.g. as much as 22% of genome size variation between two lines of maize mainly caused by TE differences [26] or a 30% increase in genome size in the Australian rice *Oryza australiensis* being caused by the recent activity of just three TE families [27]. However, because of their repetitive nature, it is generally challenging to follow the evolutionary fate of individual TE copies as soon as divergence increases. Hence, the limitation of this “global” approach is that it has limited power to pinpoint factors that prevent or promote the invasion of TEs within a given genome [13, 28, 29].

The Arabidopsis genus is a model of choice to study the dynamics of TEs [30]. Deep annotation by Maumus and Quesneville [23] of the repeated fraction of the high quality genome assemblies of the selfer *A. thaliana* [2] and the outcrosser *A. lyrata* [31] revealed that the fraction of the genome with substantial similarity to TE sequences was more important than previously appreciated, and consisted of two distinct populations of TE sequences. Beside a large number of sequences of short, likely degraded TE-derived sequences with an ancient insertion history in both genomes, there is a massive population of recently inserted TEs in *A. lyrata* (inserted within the last million years), which is largely absent from the *A. thaliana* genome [23]. The presence of TEs is associated with reduced levels of gene expression for TEs up to 2.5 kb away in *A. thaliana,* while in *A. lyrata* TEs as close as 1 kb are not associated with reduced expression of the nearby gene [32, 33]. Furthermore, He et al. [34] observed in F1 hybrids a consistent bias of TE transcript levels towards the *A. lyrata* copy, suggesting that *A. lyrata* TEs are less efficiently silenced than their *A. thaliana* orthologs, possibly as a result of differences in the methylation control machinery between the two species [30]. However, a comprehensive understanding of TE evolution in the Arabidopsis genus is difficult to obtain from this comparison alone [23], especially since the difference in mating system between the two species constitutes an important confounding factor [35–37].

To obtain a more general picture of how TEs evolve in the model Arabidopsis genus, it is thus essential to compare species with identical mating systems. To follow the evolutionary fate of individual TE copies, we studied the divergence of the TE repertoires of two closely related outcrossing species, *A. lyrata* and *A. halleri* that diverged less than 1 million years ago [38], including at an even finer scale the comparison between the subspecies *A. halleri halleri and A. halleri gemmifera.* These (sub) species remain phylogenetically close enough that TE insertions can be tracked individually. We find that these genomes host an abundant population of recently inserted TEs with almost identical insertion ages, although only a very small fraction are found at orthologous positions, indicating a very rapid turnover of these sequences. The small fraction of TE-derived sequences that is retained over the long run displays distinctive features, with gene proximity an important factor favoring TE retention. We argue that while TE accumulation in genomes has typically been studied in light of the dynamics of new insertions, their propensity for long-term retention by resisting deletion is also an important factor.

## Results

### Comparing and improving genome assemblies in outcrossing Arabidopsis species

**Fig. 1:**
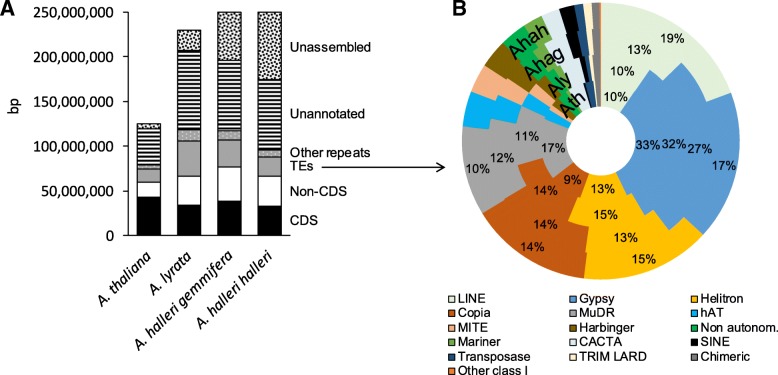
Genome composition and detailed TE content of the four assemblies. **a** the genomes are represented as vertical bars, split up by annotation type. For clarity, bases belonging to more than one category (overlapping annotations such as TEs included in genes) were discarded from the figure (1.84% of the total assembly size overall). Genome size estimates are from flow cytometry experiments [39, 46]. **b** Relative coverage of each TE family

To compare TE repertoires in outcrossing Arabidopsis species, we used the high quality Sanger-based *A. lyrata* genome assembly [31], and the recently published genome assembly of the Asian subspecies *A. halleri gemmifera* [39]. To improve contiguity of the recent genome assembly of *A. halleri halleri* [40], we produced additional Illumina paired-end and mate pairs as well as PacBio sequencing reads (Additional file 1)*.* We sequenced a total of (i) 12,560,731,806 base pairs using Illumina sequencing (~48x coverage of the genome) and (ii) 4,713,108,471 base pairs (~18x coverage) using PacBio sequencing with an average subread size of 3332 bp. A new Illumina-based assembly was produced combining the new reads and the reads of [40], and the PacBio long reads were used for scaffolding leading to a substantial 3-fold decrease of the number of scaffolds (9891 to 3152) and a 5-fold increase of the N50 (52 kb to 279 kb, Table 1). Although the resulting assembly remains more fragmented than the *A. halleri gemmifera*, *A. lyrata* and *A. thaliana* assemblies we used in this study both in terms of the number of scaffolds and a lower N50 (Table 1), the fraction of coding sequences was roughly comparable, with only slight variations in the proportion of genic non-CDS sequences and shorter genic non-CDS sequences in *A. thaliana* (Fig. 1a), as noted previously [31]. Furthermore, the higher fragmentation of the assembly affected only slightly the representation of the coding genes, since quantitative measures for the assessment of the different assemblies using BUSCO [41] showed similar numbers with only 46 of the 1440 universally conserved plant genes missing from the *A. halleri halleri* assembly (3.2%) vs. 17 in *A. lyrata* (1.2%*,* Table 1).

**Table 1:** Summary metrics of the four assemblies showing the relative levels of completeness and fragmentation

	*A. halleri halleri*	*A. halleri gemmifera*	*A.lyrata*	*A. thaliana*
Nb scaffolds	3152	2239	695	7
Total length	174 Mb	196 Mb	207 Mb	120 Mb
Genome cov.	68.3%	76.9%	89.9%	88.9%
Longest scaff.	1.5 Mb	4.3 Mb	33.1 Mb	30.4 Mb
N50	279,389	712,249	24,464,547	23,459,830
L50	177	71	4	3
CDS content	18.7%	19.5%	16.3%	35.4%
non-CDS gene content	19.3%	19.6%	15.9%	14.9%
Number of TEs	68,583	85,835	87,477	39,210
TE content (TE content estimation)	15.2% (32.7%)	20.8% (30.2%)	27.8% (24.5%)	17.4% (19.1%)
Other repeats content	4.5%	5.4%	5.8%	4.3%
Unannotated bases	42.2%	34.8%	34.7%	28.1%
Complete universal single-copy orthologs	95.3%	97.6%	98.5%	98.2%
Fragmented universal single-copy orthologs	1.5%	0.3%	0.3%	0.5%
Missing universal single-copy orthologs	3.2%	2.1%	1.2%	1.3%

### Orthology map of genes in the assemblies

The orthology relationships between genes were defined using inParanoid [42]. We identified 16,702 inparalog and ortholog clusters for *A. halleri halleri* and *A. lyrata*, in agreement with figures obtained using transcriptome data [43]. After removing clusters containing paralogs and applying stringent criteria (see methods), we conserved 15,620 orthologous genes between these two assemblies i.e. 57.5 and 47.8% of the total number of annotated genes in *A. halleri halleri* and *A. lyrata*, respectively. Reciprocal best hit Blastp approach between translated CDS of the two species led to similar results with a total of 16,900 orthologous genes (identity ≥85%, coverage of the query and the subject ≥60%). Similar numbers of inparalog and ortholog clusters were identified for human and chimpanzee using a comparable approach [44]. Using the same procedure, we identified 17,705 and 15,240 orthologous genes for *A. halleri gemmifera* and *A. lyrata*, and for *A. halleri halleri* and *A. halleri gemmifera*, respectively.

### Identifying and annotating TEs

To minimize bias due to annotating TEs using sequences from different reference genomes, we built libraries of consensus sequences that are representative of repetitive elements identified in each assembly separately using the *TEdenovo* pipeline of the package REPET [23]. The libraries were then pooled to form a “bundle” library. Each consensus sequence was classified into types of repeats and TE superfamilies using PASTEC [45]. Finally, the bundle library was used to annotate TEs in each assembly in parallel. Overall, the bundle library was composed of 3821 families of repeats. This library was used to annotate 68,583; 85,835; 87,477 and 39,210 TEs in *A. halleri halleri*, *A. halleri gemmifera*, *A. lyrata* and *A. thaliana*, respectively (Table 1). Our deep repeatome annotation strategy thus confirmed the higher proportion of TEs in *A. lyrata* (27.8%) than in *A. thaliana* (17.4%), as previously noted by Maumus and Quesneville [23]. Taken at face value, the proportion of TEs in *A. halleri halleri* and *A. halleri gemmifera* appears lower than in *A. lyrata* (Table 1), but this is probably not the case since these two assemblies are markedly less complete and a substantial proportion of the unassembled genome probably corresponds to repeats. To overcome this problem, we mapped the raw sequencing reads onto the bundle library, which provides an estimate of the proportion of TEs that is assembly-independent. Using this approach, we estimated that 32.7 and 30.2% of the *A. halleri halleri* and *A. halleri gemmifera* genomes are composed of TEs, with lower proportions in *A. lyrata* (25.2%) and the lowest proportion in the *A. thaliana* reference genome Col-0 (19.1%). Hence, we confirm that the three outcrosser species *A. lyrata*, *A. halleri halleri* and *A. halleri gemmifera* genomes have higher TE content than the selfer *A. thaliana*, consistent with the slightly larger genome size of *A. halleri* as compared to *A. lyrata* based on flow cytometry [46].

Within the TE fraction, the relative proportion of the major superfamilies were roughly comparable, with Gypsy, Copia, LINE, MuDR and Helitron as the five most abundant superfamilies in all genomes, although their relative ranking varies (Fig. 1b). Hence, the higher abundance of TEs in *A. lyrata*, *A. halleri halleri* and *A. halleri gemmifera* as compared to *A. thaliana* is not due to just one TE family having expanded but rather to a more general process of accumulation over several families.

### Age distribution of TEs

**Fig. 2:**
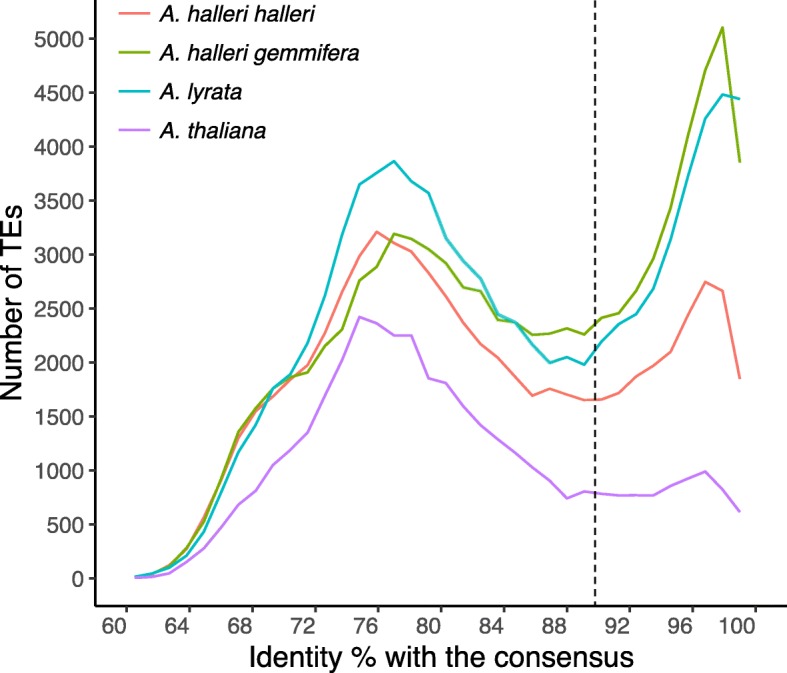
Distribution of nucleotide identity of TEs to the consensus sequence of their TE family for the three species. This statistic is used as a proxy for the relative age of TE insertion. Based on this distribution, we define “old” and “young” TEs based on a threshold of 90% identity represented by the dashed vertical line (close to the lowest point of the distribution)

The distribution of the values of identity of individual TEs to the consensus sequence of their family (classically taken as a proxy for the relative age of their insertion since TEs are initially fully identical to their copy of origin [47], but see [48]) shows that the two clearly distinct populations of TEs observed in *A. lyrata* [23] are also observed in *A. halleri halleri* and *A. halleri gemmifera*. Specifically, the distribution of percentage of identify was clearly bimodal with as many as 21,160 (30.9% of the total number of TEs) and 35,010 (40.8%) TEs with over 90% identity in *A. halleri halleri* and *A. halleri gemmifera,* respectively (Fig. 2)*.* In the following, we define “recent” vs “old” TEs in relation to this 90% threshold. The distribution profile in *A. halleri gemmifera* is very similar to the one observed in *A. lyrata (n = 32*,318 i.e. 36.9%) but the peak of very similar TEs is less pronounced in *A. halleri halleri*, possibly due to differences in the quality of the assembly for the most recent copies. In contrast, the number of TEs with over 90% identity was lower in *A. thaliana* (*n* = 7938 i.e. only 20.2% of the total number of TEs, Fig. 2), confirming the sharply different age distribution of TEs in this species [23]. Hence, the peak of putatively recent TEs observed in the *A. lyrata* genome is also observed in *A. halleri halleri* and *A. halleri gemmifera*.

**Fig. 3:**
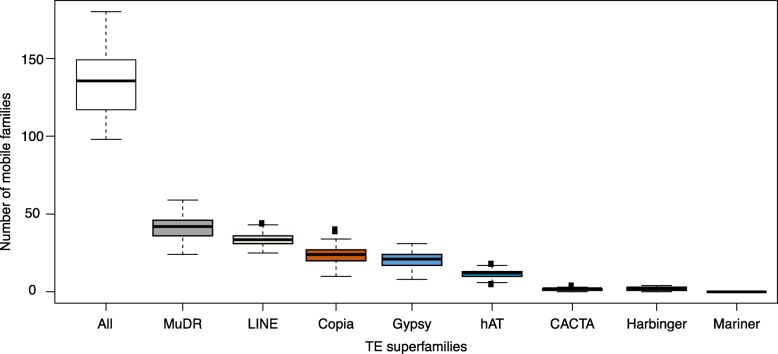
Mobilome composition and variation among *A. halleri gemmifera* accessions

The different TE superfamilies differed in their contribution to the peaks of recent and ancient TEs. The LINE superfamily, for instance, had very low contribution to the recent peak, while the other four (Gypsy, Copia, MuDR and Helitron) had sometimes very sharp peaks of recent TEs (Additional file 2). Moreover, the peak of recent TEs is not caused by any single TE superfamily, but rather corresponds to the recent activity of several TE superfamilies, and hence corresponds to a general TE mobilization phenomenon. In order to evaluate the current dynamics of mobilization, we used a recently developed approach [22] based on the mapping of short Illumina reads from multiple individuals to detect segregating insertions that are not present in the reference assembly and show the hallmark of their recent transposition (presence of the target site duplication, TSD). We found that the superfamily composition of this set of presumably currently active copies in 54 *A. halleri gemmifera* individuals [49] is very similar to that of the peak of recent TEs present in the assembly (Fig. 3 and Additional file 2). Hence, TE mobilization appears to be ongoing and the relative contribution of the different superfamilies seems to have remained relatively stable in the recent past.

### Low proportion of orthologous TEs in spite of recent divergence

**Fig. 4:**
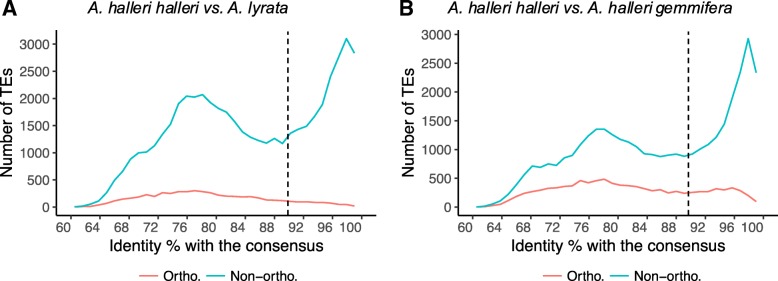
Distribution of nucleotide identity of TEs to the consensus sequence of their TE family. **a**: comparison between *A. halleri halleri* and *A. lyrata*, **b**: comparison between *A. halleri halleri* and *A. halleri gemmifera.*

Next, we sought to follow the fate of individual TE copies between pairs of lineages in order to distinguish TEs that have been either specifically inserted or deleted in one of the two lineages from TEs that have been maintained at orthologous positions since the divergence between pairs. To define TE orthology in a context where the compared assemblies exhibit different levels of contiguity, we used stringent positional information determined by the identity of the pair of flanking genes with a strict one-to-one orthology relationship between the two genomes compared. The presence of a TE in the orthologous intergenic interval was then determined based on a relaxed Blast search procedure. For TEs within genic sequences, we searched for the presence of a TE in the unambiguous ortholog, when it existed. In turn, to avoid spurious results due to multiple hits that may arise because of the relaxed Blast criteria, we restricted the analysis to orthologous intergenic segments shorter than 70 kb and discarded TEs that are either on contigs with no orthologous gene or on the extremity of contigs. We also required that the hits belong to the same TE cluster in the bundle library, although relaxing this criterion did not affect our results qualitatively. Using this set of conditions, the intergenic segments considered contained an average of 2.7 distinct TE sequences, thus enabling us to cross-check their presence (orthology) and absence (non-orthology) in the two genomes with good accuracy. In spite of the use of relaxed Blast parameters, our analysis identified only 5273 orthologous TEs between *A. halleri halleri* and *A. lyrata*, representing a minority of the TEs. Specifically, this number of orthologous TEs represents 20.3% of the 25,990 TEs in *A. halleri* and 14.7% of the 35,798 interrogated TEs from *A. lyrata* (Fig. 4a). As expected, a higher proportion of orthologous TEs was detected when comparing the very closely related *A. halleri halleri* and *A. halleri gemmifera (*i.e. at the sub-species level, 41.8 and 29.5%; Fig. 4b).

The age distribution of orthologous TEs (as defined by the divergence to their consensus) was strikingly different from that of non-orthologous TEs. As expected, orthologous TEs were almost exclusively old with a very small fraction belonging to the population of recently inserted TEs, whereas non-orthologous TEs were either anciently or recently inserted, with a relative proportion of these two categories closely matching that of the overall genome (Fig. 4, Table 2). Similar results were observed in the comparison between *A. halleri gemmifera* and *A. lyrata* (Additional file 3). Given the time scales considered, recently inserted TEs may either have inserted after the species became isolated, or have been present in the ancestor some time before the split and have been removed in one of the two species. It is therefore impossible to unambiguously reconstruct their evolutionary history.

**Table 2:** Proportion of orthologous and non-orthologous TEs in pairwise comparisons

Pairwise comparison	*A. halleri halleri* vs *A. lyrata*	*A. halleri halleri* vs *A. halleri gemmifera*
Total orthologous TEs	5273	9911
Old orthologous TEs	4642	7634
Young orthologous TEs	631	2277
Total non-orthologous TEs	51,242	37,534
Old non-orthologous TEs	31,471	21,270
Young non-orthologous TEs	19,771	16,264

### Factors associated with long-term maintenance of ancient TEs

**Fig. 5:**
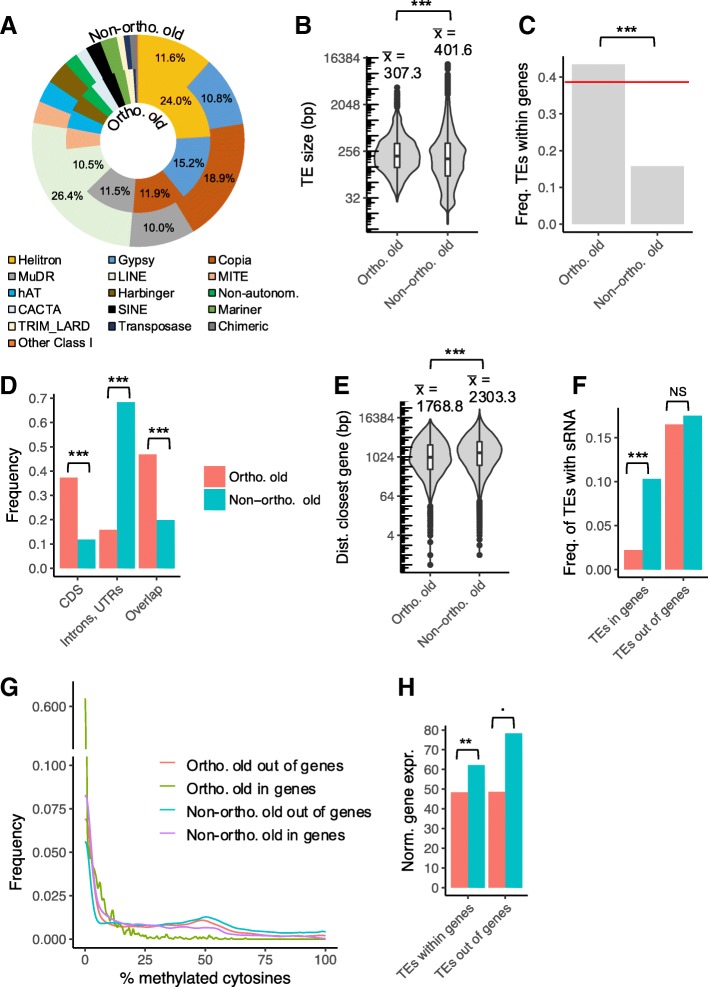
Identification of factors related to the long-term maintenance of old TEs using the comparison between *A. halleri halleri* and *A. lyrata.* **a** superfamily composition, **b** TE length, **c** frequency of orthologous and non-orthologous TEs within genic sequences. The red line indicates the proportion of genic sequences in the *A. halleri halleri* assembly, **d** frequency of orthologous and non-orthologous TEs within different categories of genic sequences, **e**: distance to the nearest gene for TEs outside of genes, **f** frequency of TEs with active siRNA production, as defined by the presence of at least 5 reads and siRNA reads covering at least 10% of the total length of the TE sequence **g** frequency of TEs related to the percentage of methylated cytosines, **h** normalized gene expression for genes containing a TE or genes without a TE. Statistical significance is indicated using the following code: “***” for *p <* 0.001, “**” for *p* between 0.001 and 0.01, “*” for *p* between 0.01 and 0.05, “.” for *p* between 0.05 and 0.1 and “NS” for *p* > 0.1

**Fig. 6:**
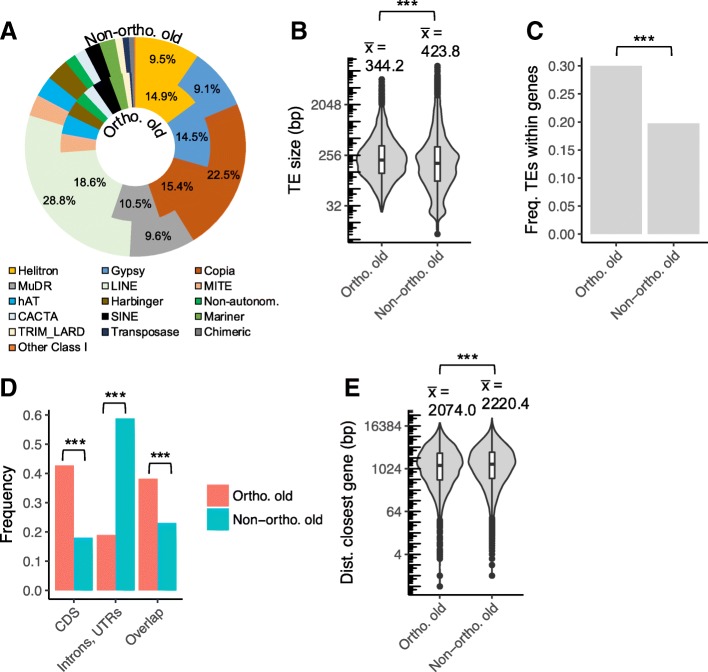
Identification of factors related to the long-term maintenance of TEs using the comparison of the TE content between *A. halleri halleri* and *A. halleri gemmifera.* **a** superfamily composition, **b** TE length, **c** frequency of orthologous and non-orthologous TEs within genic sequences, **d** frequency of orthologous and non-orthologous TEs within different categories of genic sequences, **e** distance to the nearest gene for TEs outside of genes. Statistical significance is indicated using the following code: “***” for *p <* 0.001, “**” for *p* between 0.001 and 0.01, “*” for *p* between 0.01 and 0.05, “.” for *p* between 0.05 and 0.1 and “NS” for *p* > 0.1

In contrast, reconstructing the evolutionary history of “old” TEs (< 90% sequence identity to their respective consensus, Fig. 4) is relatively straightforward. We note that, by definition, this population of TE-derived sequences has accumulated mutations since their insertion, and so corresponds to largely degraded and likely inactive copies rather than full-length elements. Assuming identical rate of divergence, old TEs had to be present in the ancestor species, so that their absence in one genome can be readily interpreted as resulting from a deletion process. Based on this assumption, we sought to identify the factors associated with long-term maintenance of individual old TEs. First, we compared the different superfamilies and found that old members from the Helitron superfamily were preferentially maintained in the long-term relative to others, since this class of TEs was more represented among orthologous than among non-orthologous TEs (24.0% vs. 11.6%, *p* < 2.2e^− 16^, Fig. 5a). Conversely, old members of the LINE and Copia superfamilies were enriched in the non-orthologous fraction (26.4 and 18.9% for LINE and Copia, respectively) relative to the orthologous fraction (10.5 and 11.9%, respectively, *p* < 2.2e^− 16^ and *p* < 2.2e^− 16^) and were therefore more rapidly deleted. Second, we found that TEs that had been maintained at orthologous positions tend to be on average 23.5% shorter than those that had been deleted from one of the two genomes (307.3 vs. 401.6 bp, *p* < 2.2e^− 16^), but the medians of the two distribution were very similar, suggesting that the difference is largely driven by a limited set of large TEs that are only found in the non-orthologous fraction (Fig. 5b). Third, we compared the location of orthologous vs. non-orthologous old TEs and observed that orthologous TEs tended to be found more often within genes than non-orthologous TEs (Fig. 5c). For instance, in the *A. lyrata* vs. *A. halleri halleri* comparison, 43.5% of orthologous TEs but only 15.8% of non-orthologous TEs were found within genic sequences (*p* < 2.2e^− 16^). The proportion of orthologous TEs in genes is close to the genomic average (genic sequences represent 38% of the overall *A. halleri* assembly, Fig. 5c), suggesting that the observed difference can be attributed to preferential removal of TEs in genic sequences (leading to disruption of orthology) rather than to preferential retention of TEs in genic sequences over the time scale examined. The same qualitative pattern was true for the *A. lyrata* vs *A. halleri gemmifera* comparison (Additional file 4) and the *A. halleri halleri* vs. *A. halleri gemmifera* comparison, albeit with a lesser contrast for the latter (30.0 vs. 19.8%, Fig. 6c). For old TEs within genic sequences, we further distinguished between TEs within CDS and non-CDS sequences. We observed that old TEs located within CDS are more likely to be retained at the orthologous state than TEs located in non-CDS sequences (Fig. 5d). In fact, around 37.3% of orthologous TEs were found within CDS sequences, while they were only 11.8% for non-orthologous TEs (*p* < 2.2e^− 16^). In line with this observation, we also observed that TEs outside genic regions tended to be retained more readily when located close to genes (Fig. 5e). Among the old TEs, those that have been retained at orthologous positions between *A. halleri halleri* and *A. lyrata* were located on average 1768.8 bp away from their closest gene, while those that have been retained either in *A. halleri halleri* or in *A. lyrata* only (and thus have been deleted from the other lineage) were located on average at a distance of 2303.3 bp, i.e. they were located 30.2% farther (*p* < 2.2e^− 16^). Overall, these results suggest that TEs in gene-rich regions tend to be protected from deletion, possibly because of the deleterious effects associated with the imprecise nature of the deletion process, which tend to remove flanking sequences as well.

We then sequenced small RNAs from the *A. lyrata* MN47 accession and compared the proportion of old TEs with substantial siRNA production (> 5 uniquely mapped reads per million reads and covering at least 10% of the length of the TE) as a proxy for efficient targeting by the RdDM pathway. As explained above, recent TEs were discarded from this analysis because of their ambiguous evolutionary history. Old TEs located within genes were less often targeted by the RdDM pathway than those outside of genes (*p* < 2.2e^− 16^) (Fig. 5f). For TEs located within genes, we found that old TEs that have remained orthologous were less likely to be RdDM targets than those that have been deleted since divergence, with 10.3% of non-orthologous TEs showing active siRNA production vs. only 2.2% for orthologous TEs (*p* < 2.2e^− 16^). However, for TEs located outside of genes we found no difference in siRNA production by orthologous vs. non-orthologous TEs (16.5 and 17.5% respectively, *p* = 0.2212). Since the mechanism of TE silencing operates through DNA methylation, we further compared the level of methylation of orthologous and non-orthologous TEs in *A. lyrata,* taking advantage of the bisulfite DNA methylation data available for *A. lyrata* [50]. In accordance with the siRNA mapping analysis, we found that orthologous TEs within genes were less methylated than any other populations of TEs (*p* < 2.2e^− 16^, Fig. 5g). Indeed, they presented a mean percentage of methylation of only 3.7% compared to 22.8, 17.9, 32.4% for non-orthologous TEs within genes, orthologous TEs outside of genes, and non-orthologous TEs outside of genes, respectively. These results suggest that a low siRNA production and low DNA methylation levels are associated with the long-term maintenance of old TEs within genes. In contrast, these two factors may not be related to the long-term maintenance of TEs outside of genes.

Finally, we used RNA-seq data from the same *A. halleri halleri* accession to compare the expression of genes containing old TE sequences that have been either retained or removed since the separation of the two species. We reasoned that if TEs are deleterious on average, removing them should be advantageous even in the face of the deleterious effect of local deletions. If so, TE removal should occur more readily close to or within genes with high expression than genes with low expression [32]. Accordingly, we found that on average genes with an orthologously-maintained TE in their DNA sequence were expressed at slightly lower levels than the genes with a TE that has been removed from *A. lyrata* (*p =* 0.006995, Fig. 5h). Furthermore, for TEs in intergenic regions the expression of the closest gene along the chromosome also tended to be lower when the TE was orthologously maintained than when it had been removed from *A. lyrata* (*p =* 0.05789).

### Genes containing non-orthologous TEs seem to be more essential

**Fig. 7:**
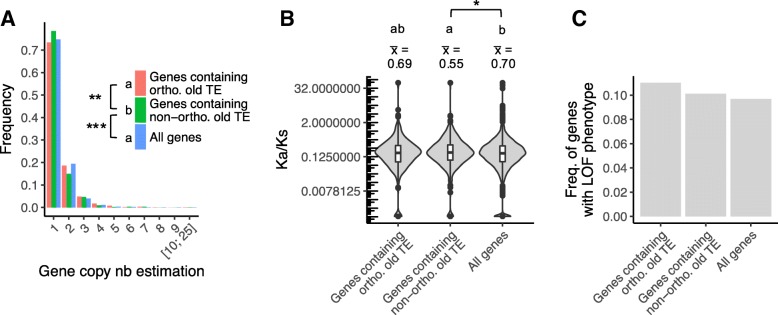
Gene essentiality of *A. halleri halleri* genes containing TE fragments that have or don’t have an orthologous copy in the *A. lyrata* genome. **a** Distribution of the size of the gene families, obtained from the estimated number of paralogs (single copy genes belong to families of size 1), **b** Ka/Ks, **c** Proportion of genes with a loss-of-function mutant phenotype. In **a** and **c**, genes containing at least one orthologous and genes containing at least one non-orthologous old TE are compared to all genes annotated in the *A. halleri halleri* genome assembly. In B, these categories are compared to all orthologous genes between *A. halleri halleri* and *A. lyrata,* since sequences from both orthologous genes are required to calculate Ka/Ks*.* Statistical significance is indicated using the following code: “***” for *p <* 0.001, “**” for *p* between 0.001 and 0.01, “*” for *p* between 0.01 and 0.05, “.” for *p* between 0.05 and 0.1. Only significant values are shown

We used several proxies of gene essentiality, including the size of the gene family (single copy genes tend to be more essential because of the lack of functional redundancy), Ka/Ks (lower values are expected for more essential genes) and the presence of a detectable loss-of-function mutant phenotype in *A. thaliana*. Results were compared across three sets of genes: all genes together vs. genes containing an orthologous TE and genes containing a non-orthologous TE. Overall, genes with non-orthologous old TEs (hence corresponding to TE deletions) tend to be more essential. First, they are more often single copy genes (78.5% compared to 74.7% for all genes, *p* = 0.0003322, and compared to 73.4% for genes containing an orthologous old TE, *p =* 0.009662, Fig. 7a). Second, they presented on average lower Ka/Ks values (0.55 compared to 0.71 for all genes, *p* = 0.028, and compared to 0.69 for genes containing an orthologous old TE, *p* = 0.422, Fig. 7b). These results suggest that TEs that have been removed in *A. lyrata* since the divergence from *A. halleri* occurred in genes that could be more essential than those in which TEs were retained, which were comparable to the average of genes. Possibly, their presence presented a more deleterious impact that could counterbalance the deleterious impact of their removal. However, the proportions of genes with a loss-of-function mutant phenotype was independent from the presence of orthologous or non-orthologous TEs (Fig. 7c).

## Discussion

### Dynamics of TE accumulation in two outcrossing species

Overall, the population of TEs in the two *A. halleri* assemblies that we studied is very similar to the one described in *A. lyrata*, both in terms of TE families present and in their age distribution. As noted previously, these profiles are sharply distinct from that seen in *A. thaliana* [23, 31, 51, 52]. This contrast has been attributed either to differences in the mating system [37, 53] or to a specific burst along the outcrosser lineages [30, 34]. Although our analysis cannot formally distinguish between these two possibilities at this stage, our results unambiguously demonstrate that the dynamics of TE accumulation that is shared between *A. halleri* and *A. lyrata* has been in place at least since their divergence, ca. 1 Myrs ago and is therefore not an event of the very recent past.

This dynamic is first characterized by the fact that multiple TE families are currently active. The young population of TEs observed in *A. halleri* and *A. lyrata* is composed of several families, with Helitron, Gypsy, MudR and LINE being the most contributing families. This is confirmed by the analysis of segregating (neo) insertions that are absent from the reference assembly in *A. halleri gemmifera*. Overall this pattern is similar to what was observed in the *A. thaliana* genome [22], albeit to an even greater scale and comes in stark contrast to the human genome, where only a few TE families contain mobile copies, all belonging to the LINE-1 and SINE families [54]. Identifying the factors causing multiple vs. just a few TE families to spread in any given genome is clearly a stimulating challenge for future TE research. In spite of the recent divergence between the two species we find very few orthologous TEs between *A. halleri* and *A. lyrata*, even for the population of “older” TE-related sequences that must have been present before speciation. Overall, even though the dynamics of TE accumulation seems to be shared, the resulting TE fractions of the two genomes are very different, indicating a rapid turnover of TE-related sequences. It will now be essential to compare quantitatively the rate at which TEs transpose and get removed between different species and how these rates are affected by various biological features. TEs have been used as phylogenetic markers in other taxa (e.g. birds [55]), where the rate of turnover of TE-related sequences seems to be slower. The rate of DNA loss varies extensively across species [56], but the determinants of this variation are poorly understood. Whether the rate and pattern of TE removal differ from the more general process of non-coding DNA loss across the genome is an important question for the future.

### Factors associated with TE deletion or maintenance

Given the very rapid elimination of old TEs that we observe, how can a substantial number of old TEs be maintained for a long period of time, while a complete elimination would have been expected if this was a continuous process? We found marked differences in the propensity of TE-related sequences to resist deletion and therefore be maintained over a time scale of ca. one million years of total divergence.

#### Long-term maintenance of helitrons, rapid removal of LINE and Copia

First, our analyses suggest that Helitron elements are more likely to be maintained over the long-term in *A. halleri* and *A. lyrata*. As noted by Maumus and Quesneville [23], helitrons tend to have lower GC content than the other TE superfamilies, which may be associated with reduced targeting by RDdM because less cytosines are available for methylation, hence leading to disruption of neighbouring gene expression. It is tempting to speculate that the lower GC content of helitrons may make them less deleterious, allowing for their preferential long-term maintenance. In contrast, LINE and Copia families are those that have been the most strongly eliminated since the divergence of the two species. Mao and Wang [57] recently observed that in grass, SINE families were retained over the long term. Like in grass, SINEs have low abundance in the *A. halleri* and *A. lyrata* genomes, since they cover less than 3% of the repeat sequences. However, in these species they do not seem to be associated with a long-term maintenance, as they are equally represented amongst old TEs that have been maintained at orthologous positions and amongst those that have been deleted from one of the two genomes (Fig. 4). Hence, the long-term maintenance of particular TE families seems to be lineage-specific and cannot easily be generalized.

#### Sheltering of TEs by proximity to genes

A striking observation is the long-term retention of TE sequences in gene-rich regions. As we focused on the population of “old” TEs that had to be present in the most recent common ancestor, this pattern is unlikely to be caused by an insertion bias of recent specific insertions towards genic regions and rather reflects a process of differential retention. Alternatively, this pattern may also be caused by gene-rich regions being better assembled, resulting in TEs in those regions being more readily found in the different assemblies. This effect is likely minor because our analysis focuses on old TEs, which should be relatively less problematic in terms of assembly because they tend to be less identical across copies. Also, in this case the most poorly assembled genomes should show less non-orthologous TEs, while here the reciprocal analyses provide similar results. The *A. lyrata* genome sequence is a high quality assembly obtained using the Sanger technology, yet does not show a specifically elevated fraction of non-orthologous TEs. Clearly, long-read technologies should resolve this issue [58].

The relative enrichment of orthologous TEs in genic sequences as compared to non-orthologous TEs is consistent with the interpretation that TEs within gene-rich regions benefit from a “sheltering” effect, whereby a deletion of the TE sequence involves the risk of also deleting part of the gene sequence, which would be highly deleterious in particular when they have become integrated within coding sequences. Hence, the effective rate of deletion might be higher for non-genic TEs than for genic TEs, resulting in a long-term enrichment of the sheltered genic TEs. This process of differential retention was less pronounced when comparing the more closely related *A. halleri halleri* vs. *A. halleri gemmifera* species, indicating that such differential enrichment is a relatively slow process. In grass, Mao and Wang [57] showed that members of the SINE TE family are often shared across species and are also enriched in and near protein coding genes, possibly as a result of differential removal of SINE copies in gene-poor regions.

#### TE deletion: a cure worse than the disease?

Our results suggest that several factors can affect the long-term retention of transposable element sequences, and in particular the proximity to highly expressed genes. We propose that the process of differential TE retention results from the balance between the deleterious effects of the TE itself and that of the deletion removing it. While the presence of TE sequences was shown to equally frequently increase or decrease gene expression [59] or to have no direct causal effect [23] in *A. thaliana* (but see [60]), we found that the more highly expressed genes rarely retain orthologous TEs. In line with Hollister and Gaut [32], this suggests that selection in favor of TE deletions varies according to the level of gene expression, with deleterious effects of TE presence generally outweighing the cost of their eventual deletion when they are close to highly expressed genes.

Earlier studies have shown that the rate of DNA loss can be highly heterogeneous across genomes [56]. It is possible that the level of sequence identity among repeated sequences may contribute to this variation, as more identical sequences are more likely to be involved in the heterologous recombination that is believed to be responsible for DNA deletions. If so, the most recently inserted TEs would be expected to show an even faster elimination, as proposed by Maumus and Quesneville [61]. This might also contribute to decrease the proportion of young orthologous TEs. Beside the fact that they might have been inserted after the species divergence (but as we explained, precisely dating these events is challenging), they might be eliminated even more rapidly than old TEs that recombine less easily. Hu et al. [31] suggested that the *A. thaliana* genome is characterized by ongoing positive selection on deletions, favoring genome shrinkage (but see [62]). It would be interesting to determine how many of these deletions involve the removal of TE sequences.

In addition to this “sheltering” effect of TEs considered as deleterious or quasi neutral elements, it is also possible that those TEs that are retained in the long term have acquired a functional beneficial role for their host genome (being “domesticated”), thus making their removal deleterious in itself. It is unclear how frequent this phenomenon might be, but several clear examples of such domestication have been reported in the literature, including the regulation of stress-response genes by acquisition of response elements carried by some TEs [13, 63] or the production of siRNAs that trigger the trans-silencing of active relatives and therefore contribute to immune memory [64–66]. A recent study however showed that TE exaptation for regulatory function is rare, and is mostly associated with “old” TEs, suggesting a model in which TE-derived sequences are initially repressed, after which a small fraction acquires and retains enhancer activity [67]. Clearly, among the repeat sequences, the old orthologous TEs that we identified here are the most likely to have acquired advantageous biological functions. Better understanding the variety of factors causing differences in retention propensity will now be an exciting and interesting next step.

## Conclusions

The comparison of whole genome assemblies of *A. lyrata* and two *A. halleri* subspecies provides an opportunity to investigate the dynamics of TEs without the confounding factor of the mating system. The time scale considered is neither too low (with no TE activity) nor too high (with complete erosion) and allows us to tease out contributing factors associated with the retention of TEs across the genome. We find that diverse TE families contribute to the ongoing dynamics of TE accumulation in the two species. TE fragments that have been maintained in both species are not a random subsample, as they tend to be located closer to genes, produce fewer short interfering RNAs, be less heavily methylated and be found within or adjacent to genes with low expression levels than those that have not resisted deletion. Our results indicate a rapid evolutionary dynamics of the TE landscape in these two outcrossing species, with an important input of a diverse set of new insertions with variable propensity to resist deletion.

## Methods

### *A. halleri* genome de novo assembly

Assembling genomes of outcrossing organisms is a challenging task because outcrossing involves a high level of heterozygosity. To increase contiguity of the recently released *A. halleri halleri* assembly based on Illumina reads [40], one paired-end (PE) and two mate-pair (MP) additional libraries were prepared from the same accession PL22-1A with the TruSeq PCR-free and the Nextera DNA library prep kits (Illumina, California, United States), respectively (Additional file 1). We additionally produced PACBIO sequences (6 SMRT cells). Quality of the Illumina raw reads was assessed using FastQC [68] (version 0.10.1), and reads were filtered accordingly using Trimmomatic [69] (version 3). When present, Ns were removed using Prinseq [70] (version 0.20.4). The total number of filtered Illumina reads ([40] and this study) represented a 110x coverage of the *A. halleri* estimated genome size [46] (~ 255 Mbp). A new de novo assembly was carried out with the AllPathsLG assembler [71] (version r44837) using all PE and MP reads. The kmer spectrum analysis carried out by AllPathsLG estimated the *A. halleri* genome size to 266 Mb, with ploidy equal to 2 and a SNP rate of 1/150, consistent with previous estimation [40]. This initial AllPathsLG assembly was then improved with the following strategy: (i) a scaffolding step was performed using the PACBIO reads and followed by a gap filling step using the PE and MP Illumina reads and (ii) a second step of scaffolding was performed using the PACBIO reads. Scaffolding was carried out using the SSPACE-LongRead.pl perl script of SSPACE [72] (version 1.1) and gap closing was achieved using the GapFiller.pl perl script of gapfiller [73] (version 1.10). Gene annotation was based on Maker [74] (version 2.31.8). EST evidence, protein homology and repeat masking references were provided from *A. thaliana*. Gene prediction was allowed from EST inference and from protein homology and resulted in the prediction of 27.992 genes. Genome metrics were obtained using QUAST [75] (version 4.0) and genome assembly and annotation completeness was assessed with BUSCO [41] (version 3) [41] using the Embryphyta odb9 dataset composed of 1440 universal single-copy orthologs.

### TE annotation

In order to produce a genome-wide annotation of repetitive sequences, the four genomes were annotated using the package REPET [76, 77] (version 2.5)*,* which is composed of two main pipelines, dedicated to de novo detection, annotation and analysis of repeats, in particular TEs, in genomic sequences (Additional file 5). Briefly, the first pipeline, TEdenovo, starts by comparing the genome with itself and clusters matches sharing at least 90% identity. Then, for each cluster, it builds a multiple alignment from which a consensus sequence is obtained. Finally, consensus sequences are classified according to TE features, and redundancy is collapsed by keeping the longest consensus from groups that share 95% of their length and 98% identity. The second pipeline dedicated to the annotation (TEannot) involves several steps, including TE detection by search for similarity between consensus and genomic sequences, the removal of hits that are included in regions corresponding to micro-and minisatellites and connection of distant fragments (up to 15 kb) using the long-join procedure [78]. In our study, a library of classified, non-redundant consensus sequences was obtained by combining the TE de novo analysis performed on the four species. Then, the bundle library was used to annotate each of the four genomes separately using TE annot.

In parallel, the proportion of TEs in each of the four genomes was estimated using an assembly-free approach. The raw sequencing reads that mapped onto the bundle library using Bowtie2 [79] (version 4.1.2) were considered as representing TEs and the other reads as non-TEs sequences. The genomic Illumina reads were obtained from [40] (37,262,746 reads in total) for *A. halleri halleri*, or downloaded from the NCBI SRA database: DRR013376 (38,782,027 reads) for *A. gemmifera*, SRR2040788 and SRR2040789 (48,602,962 reads in total) for *A. lyrata* and ERR1399719 (38,425,727 reads) for *A. thaliana*.

### TE orthology

TE orthology relationships were obtained for each pairwise comparison i.e. *A. halleri halleri* vs. *A. lyrata*, *A. halleri halleri* vs. *A. halleri gemmifera* and *A. halleri gemmifera* vs. *A. lyrata* using the orthology of genes as detailed in Additional file 6. Briefly, an orthology map of genes, using CDS annotations (excluding all CDS annotations included in TE annotations), was constructed with Inparanoid [42]. The *A. thaliana* genome was used as outgroup in the comparison of *A. halleri halleri* or *A. halleri gemmifera* vs. *A. lyrata*, whereas the *A. lyrata* genome was used as outgroup when comparing *A. halleri halleri* and *A. halleri gemmifera*. To avoid spurious hits, a stringent score cut-off of 100 bits was applied, paralogs were eliminated from the analysis, and only clusters with bootstrap values ≥99% for each of the two orthologs were conserved. Then, we selected only TEs located between two genes of this orthology map (called “framed” TEs, or TEs located within a genic sequence (“inserted” TEs). For each “framed” TE in one species, a blast search was performed between the TE sequence and the genomic sequence between the same pair of orthologous genes in the other species. We restricted this analysis to chromosomal segments of at most 70 kb (from either the subject or the query genome). We explored different values of this threshold (50 kb and 100 kb), which did not affect our results substantially (data not shown). Similarly, for “inserted” TEs, the TE sequence was compared with the orthologous gene sequence. Both “framed” and “inserted” TEs presenting a blast hit with an E-value ≤1E^− 10^, an identity ≥80% and at least some overlap with a TE annotation belonging to the same cluster family were defined as orthologous. Those that presented a blast hit with the criteria defined above but with a TE annotation from another cluster family were discarded from the analysis. The other TEs were defined as non-orthologous. These criteria correspond to a relatively relaxed search and should result in a strong power to detect orthologous TEs, resulting in a conservative analysis.

### TE analyses

Several methods have been proposed in the literature to estimate the age of TE insertions. A recently proposed approach relied on a phylogeny of individual copies within TE families [48]. This approach requires aligned sequences of TE copies, and so will be most useful for full-length TEs, or at least for copies that can be aligned over a substantial fraction of their length. In our case however, most TE sequences were short TE fragments that covered different parts of the consensus and therefore cannot be aligned, preventing proper use of the phylogenetic framework. We therefore based our age comparisons on the widely used “consensus” approach [23], whereby the values of identity of individual TEs to the consensus sequence of their family are taken as a proxy for the relative time since they started to diverge from their ancestor. Based on this metric, individual TE copies were separated into “young” and “old” classes according to whether they reached the cut-off of 90% identities with the consensus sequence of their cluster. Following [48], we note that this approach to estimate the age of insertions contains some caveats and so should be taken with caution. We further note that most of our analyses rely on orthologs comparisons based on positional information that is entirely independent from the estimation of insertion age. Differences in the representation of the different superfamilies were tested using a χ^2^ test with 1 degree of freedom. Differences in the TE size and TE distance to the nearest gene were tested using a non-parametric Mann-Withney test. Differences in the proportion of genic vs. non-genic TEs and in the proportions of CDS vs non-CDS TEs were tested using a χ^2^ test with 1 and 2 degrees of freedom, respectively.

### Identification of segregating non-reference (neo)insertions

We used a modified version of the pipeline developed by Quadrana et al. [22] to identify segregating non-reference (neo) insertions in the large population sample of 54 *A. halleri gemmifera* individuals (SRA DRA003268, omitting samples OK037001 and OK037003 because of low coverage). Basically, this pipeline was modified to consider both discordant and split-reads to call insertions. The analysis has two steps. We first performed de novo detection of non-reference TE insertions, for which put a threshold of at least ten supporting reads (discordant-reads + split-reads). We then assessed the presence or absence of these putative non-reference TE insertions across the whole population by relaxing the parameters (at least two discordant-read and/or split-read) used to detect them in the first place. This improved the discovery of putative TE-insertions that are shared by more than one accession.

### siRNA mapping and DNA methylation analyses

Total RNA was isolated from leaves of *A. lyrata* MN47 using the Qiagen miRNeasy Mini Kit (catalog #217004). A total of 3 μg of RNA was sent to LC sciences (Houston, TX, USA) were an Illumina TruSeq Small RNA library was constructed and sequenced, leading to the obtention of approximately 14 million 1 × 50 bp reads. Adaptators were removed from the Illumina reads using Cutadapt [80] (version 1.2.1) and reads were cleaned using Prinseq (version 0.20.4, Schmieder and Edwards 2011) with specified parameters: -min_len 15 –max length 25 –noniupac -min_qual_mean 25 -trim_qual_right 20 -ns_max_n 0. The quality of the Illumina cleaned reads was checked using FastQC [68] (version 0.11.4). rRNAs, tRNAs, snRNAs and snoRNAs were removed from the sRNAs sequences through Bowtie [81] (version 1.0.0) alignments using a set of 7743 eukaryotic sequences obtained from NCBI database corresponding to these types of non-coding RNAs. sRNA reads were mapped on the *A. lyrata* MN47 genome [31] using Bowtie. Multiply mapping reads were discarded and only alignments presenting no mismatch were conserved. A TE was defined as producing substantial siRNAs when it presented an overlap with more than 5 reads per million and when it was covered on more than 10% of its length. Differences were tested using a χ^2^ test with 1 degree of freedom.

The DNA methylation matrix for *A. lyrata* MN47 of Seymour et al. [50] was used to evaluate the methylation status of orthologous vs non-orthologous TEs. Following Seymour et al., we considered a cytosine site as significantly methylated when its methylation rate was ≥20% in at least one of the four tissue-treatment combinations (shoot, root, 4 °C, 23 °C). Then, we calculated for each TE the percentage of methylated sites. Differences were tested using a Kruskal-Wallis test and pairwise comparisons were performed using Tukey and Kramer test.

### Gene expression analysis

To evaluate gene expression, we generated RNA-seq data from shoot of *A. halleri* PL22-1A plants cultivated in standard greenhouse conditions. The number of reads mapped on each transcript of the PL22 reference transcriptome [43] were counted and normalized (TPM) [82]. Correspondence between transcripts from the reference transcriptome and gene models in the assembly was established by Blast using a stringent criteria (95% identity over at least 100 bp).

Differences in gene expression were tested using a Mann-Withney test.

### Proxies of essentiality of *A. halleri* genes

Size of the gene family was estimated using an all-against-all Blast approach performed from the CDS and removing hits with a query coverage inferior to 50% and/or an E-value superior to 1E^− 30^. For Ka/Ks calculation, CDSs from each pair of orthologous genes between *A. halleri* and *A. lyrata* were aligned using Water from the EMBOSS package [83] and alignments were submitted to KaKs_Calculator2.0 [84] using the Goldman and Yang method [85]. Essential genes were annotated using a dataset composed of 2400 Arabidopsis genes with a loss-of-function mutant phenotype [86]. Statistical significance was tested using a χ^2^ test for gene copy numbers and proportions of genes with a loss-of-function mutant phenotype, and using a Kruskal-Wallis test for Ka/Ks distributions. In the latter case, pairwise comparisons were performed using the Tukey and Kramer test.

## Additional files

Additional file 1: Summary statistics of input sequence data for de novo assembly of the *A. halleri* genome. (PDF 46 kb)

Additional file 2: Distribution of identity of TEs to the consensus sequence of their TE family, separated by superfamily. For each species, superfamilies are sorted according their contribution to the peaks of the most recent population of TEs (using a threshold of 98%). (PDF 91 kb)

Additional file 3: Proportion of orthologous and non-orthologous TEs in *A. halleri gemmifera* and *A. lyrata* genomes. (PDF 38 kb)

Additional file 4: Identification of factors related to the long-term maintenance of TEs using the comparison of the TE content from *A. halleri gemmifera* and *A. lyrata.* A: distribution of nucleotide identity of TEs to the consensus sequence of their TE family, B: superfamily composition, C: TE length, D: frequency of orthologous and non-orthologous TEs within genic sequences, E: frequency of orthologous and non-orthologous TEs within different categories of genic sequences, F: distance to the nearest gene for TEs outside of genes. Statistical significance is indicated using the following code: “***” for *p <* 0.001, “**” for *p* between 0.001 and 0.01, “*” for *p* between 0.01 and 0.05, “.” for *p* between 0.05 and 0.1 and “NS” for *p* > 0.1. (PDF 137 kb)

Additional file 5: Pipeline used for the deep repeatome annotation. (PDF 30 kb)

Additional file 6: Strategy to identify orthology relationships of TE sequences as determined by positional information from the flanking genes. The first step consists in defining the orthology of genes (blue squares) between genomes X and Y using Inparanoid. In our example, A/A’, C/C′ and E/E’ are considered as orthologous pair of genes (represented by blue arrows). The orthology of TEs is defined sequentially for genome X and Y but the process are similar: only TEs between two orthologous genes spaced for at most 70 kb (black squares named a and b in our example) (named “Framed”) and TEs located within genes (black square c) (named “Inserted”) are analysed. TEs which are located at an extremity of a scaffold (d and d’) and TEs located on scaffold without orthologous genes are discarded. The sequence of the TE a and b, which are located between A and C genes of the orthology map are compared using Blastn (thresholds: Evalue ≤1E^− 10^, an identity ≥80%) to the sequence between the orthologous genes of A and C, i.e. A’ and C′. The TE a presents a blast hit, and a TE annotation overlaps the Blast hit in genome Y. Hence a and a’ are defined as orthologous. No-significant blast hit is retrieved for b, which is defined as non-orthologous. The sequence of the TE c located within the E gene is compared to the sequence of the E’ gene. In our example, we considered that the Blast hit is significant and overlaps a TE annotation in Genome Y. The TE c is defined as orthologous. (PDF 11 kb)

## Data Availability

The datasets generated during the current study were deposited in the NCBI-SRA database (BioProject PRJNA495003).

## Abbreviations

CDS: Coding sequence;
piRNA: Piwi-interacting RNA
RdDM: RNA-dependent DNA methylation
siRNA: Small interfering RNA
TE: Transposable elements
