# Supplementary material for: Differential retention of transposable element-derived sequences in outcrossing Arabidopsis genomes
Source: Mob DNA. 2019 Jul 17;10:30. doi: 10.1186/s13100-019-0171-6 (PMC6636163; doi:10.1186/s13100-019-0171-6)
Supplement: Supplementary file 1 — Summary statistics of input sequence data for de novo assembly of the A. halleri genome. (PDF 46 kb) [file 13100_2019_171_MOESM1_ESM.pdf]

Library type	Library fragment size	Read size	# of reads (after filtering)
PE[1,2]	550 bp	301 bp	43,347,614
PE[1,3]	350 bp	101 bp	31,177,878
PE[2]	500 bp	301 bp	34,566,750
MP[1,2]	4 kb	301 bp	6,999,466
MP[2]	6 kb	301 bp	3,761,466
MP[2]	10 kb	301 bp	3,401,790
PACBIO	/	3.3 kb[4]	1,390,999[5]

[1]Libraries produced and sequenced by [40].

[2] Sequenced in a MiSeq Illumina sequencer (Illumina, California, United States)

[3] Sequenced in a HiSeq2000 Illumina sequencer (Illumina, California, United States)

[4] mean subread length

[5] no filtering
