# Supplementary material for: Differential retention of transposable element-derived sequences in outcrossing Arabidopsis genomes
Source: Mob DNA. 2019 Jul 17;10:30. doi: 10.1186/s13100-019-0171-6 (PMC6636163; doi:10.1186/s13100-019-0171-6)
Supplement: Supplementary file 2 — Distribution of identity of TEs to the consensus sequence of their TE family, separated by superfamily. For each species, superfamilies are sorted according their contribution to the peaks of the most recent population of TEs (using a threshold of 98%). (PDF 91 kb) [file 13100_2019_171_MOESM2_ESM.pdf]

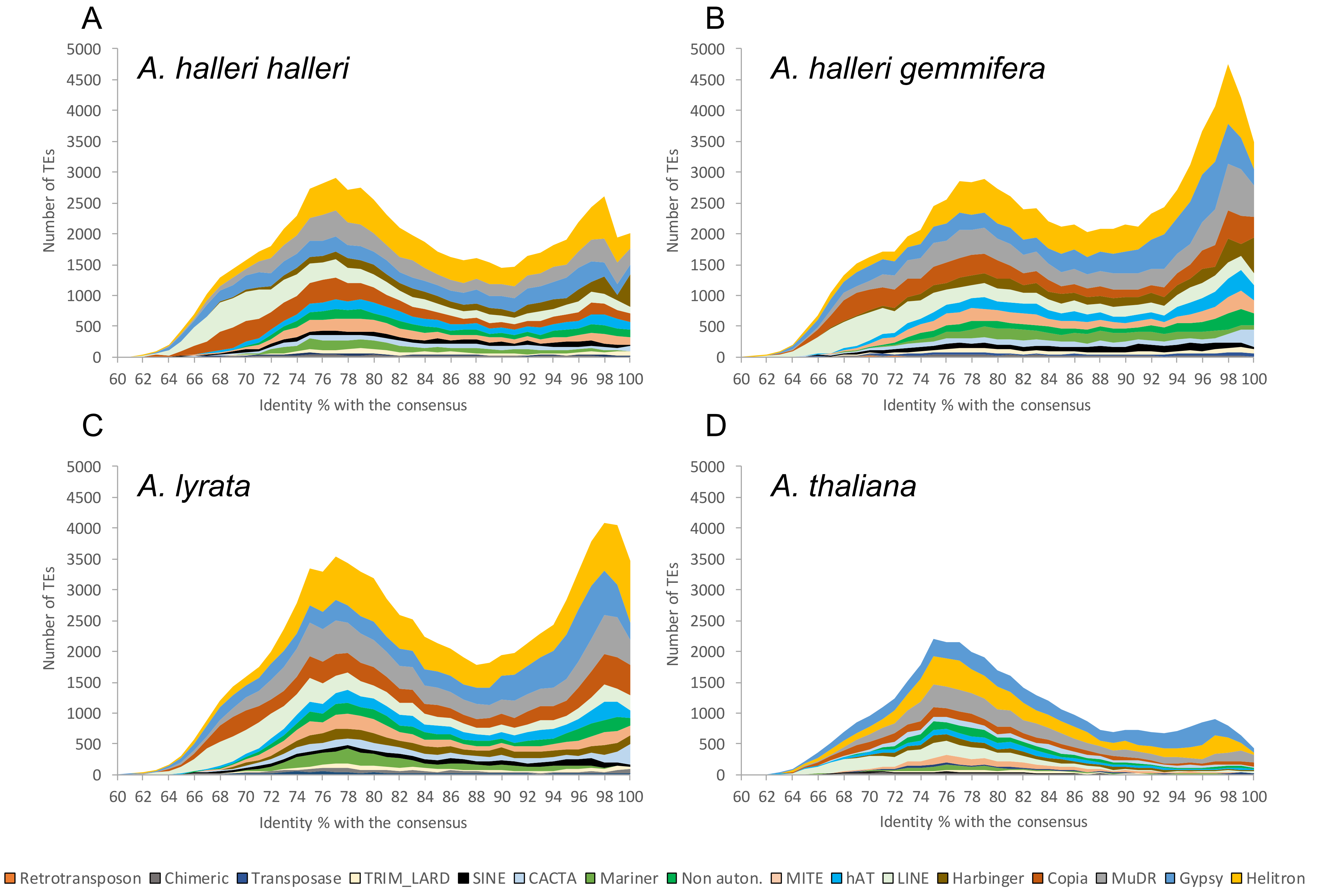
A
A. halleri halleri
B
A. halleri gemmifera
C
A. lyrata
D
A. thaliana
Number of TEs
0 500 1000 1500 2000 2500 3000 3500 4000 4500 5000
60 62 64 66 68 70 72 74 76 78 80 82 84 86 88 90 92 94 96 98 100
Identity % with the consensus
Retrotransposon Chimeric Transposase TRIM_LARD SINE CACTA Mariner Non auton. MITE hAT LINE Harbinger Copia MuDR Gypsy Helitron
