# Supplementary material for: Differential retention of transposable element-derived sequences in outcrossing Arabidopsis genomes
Source: Mob DNA. 2019 Jul 17;10:30. doi: 10.1186/s13100-019-0171-6 (PMC6636163; doi:10.1186/s13100-019-0171-6)
Supplement: Supplementary file 3 — Proportion of orthologous and non-orthologous TEs in A. halleri gemmifera and A. lyrata genomes. (PDF 38 kb) [file 13100_2019_171_MOESM3_ESM.pdf]

Pairwise comparison	*A. halleri gemmifera vs A. lyrata*
Total orthologous TEs	7,700
Old orthologous TEs	6,639
Young orthologous TEs	1,061
Total non-orthologous TEs	81,893
Old non-orthologous TEs	46,519
Young non-orthologous TEs	35,374
