# Supplementary figures and images for: Differential retention of transposable element-derived sequences in outcrossing Arabidopsis genomes
Source: Mob DNA. 2019 Jul 17;10:30. doi: 10.1186/s13100-019-0171-6 (PMC6636163; doi:10.1186/s13100-019-0171-6)

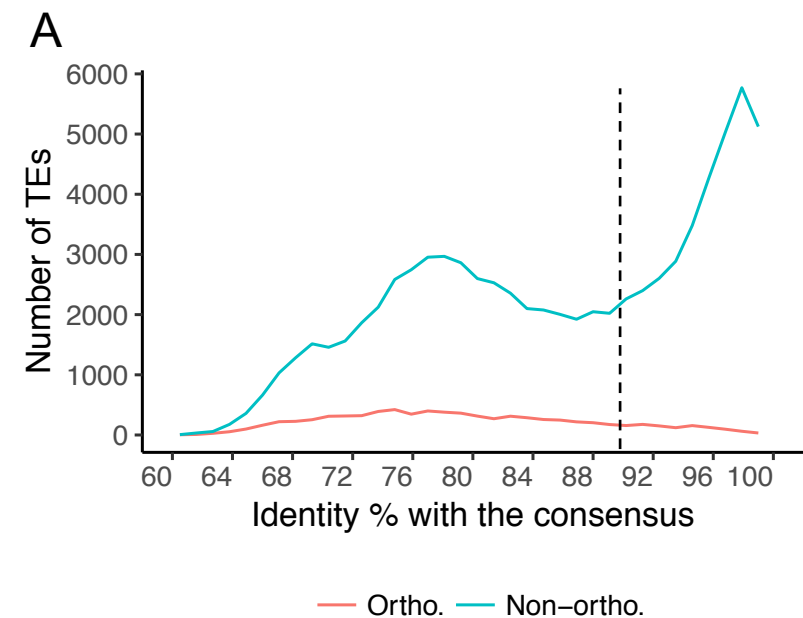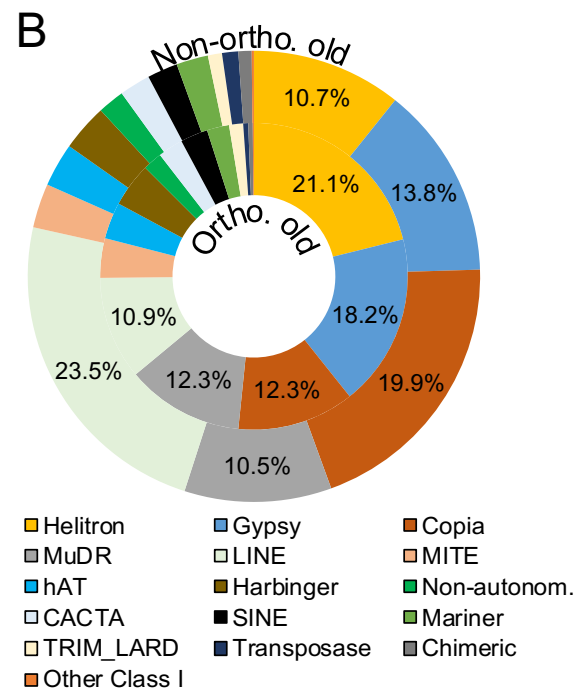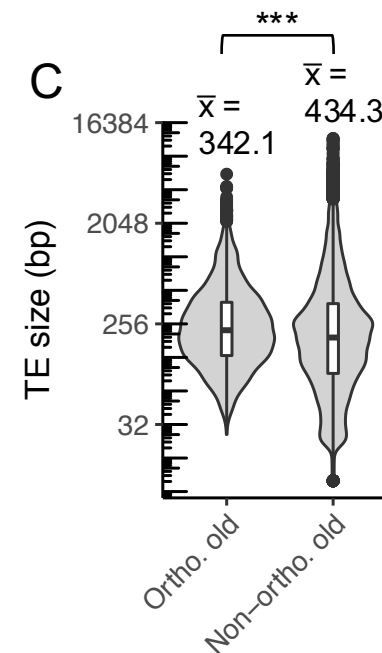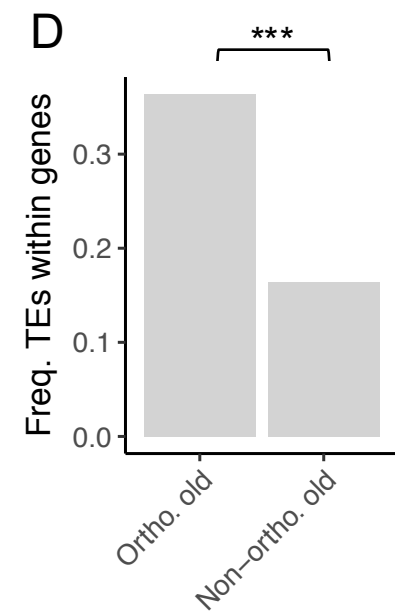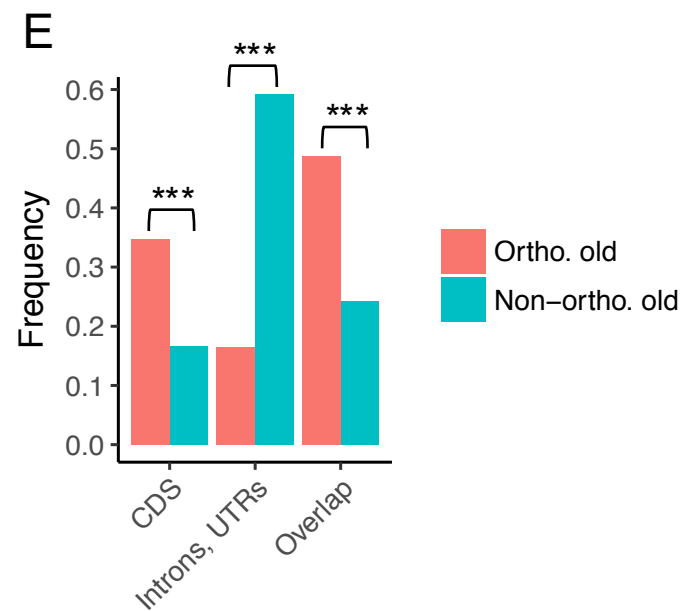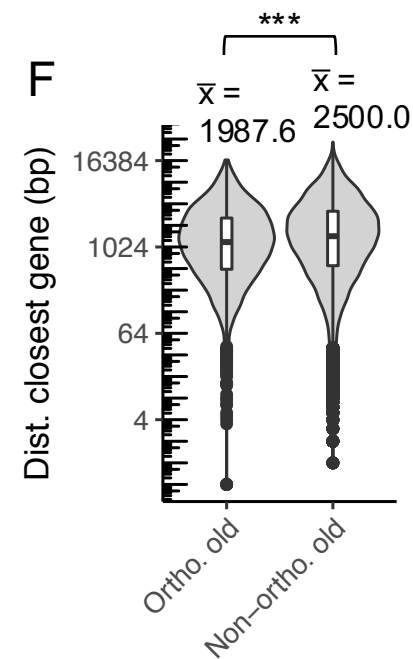

Supplement: Supplementary file 4 — Identification of factors related to the long-term maintenance of TEs using the comparison of the TE content from A. halleri gemmifera and A. lyrata. A: distribution of nucleotide identity of TEs to the consensus sequence of their TE family, B: superfamily composition, C: TE length, D: frequency of orthologous and non-orthologous TEs within genic sequences, E: frequency of orthologous and non-orthologous TEs within different categories of genic sequences, F: distance to the nearest gene for TEs outside of genes. Statistical significance is indicated using the following code: “***” for p < 0.001, “**” for p between 0.001 and 0.01, “*” for p between 0.01 and 0.05, “.” for p between 0.05 and 0.1 and “NS” for p > 0.1. (PDF 137 kb) [file 13100_2019_171_MOESM4_ESM.pdf]

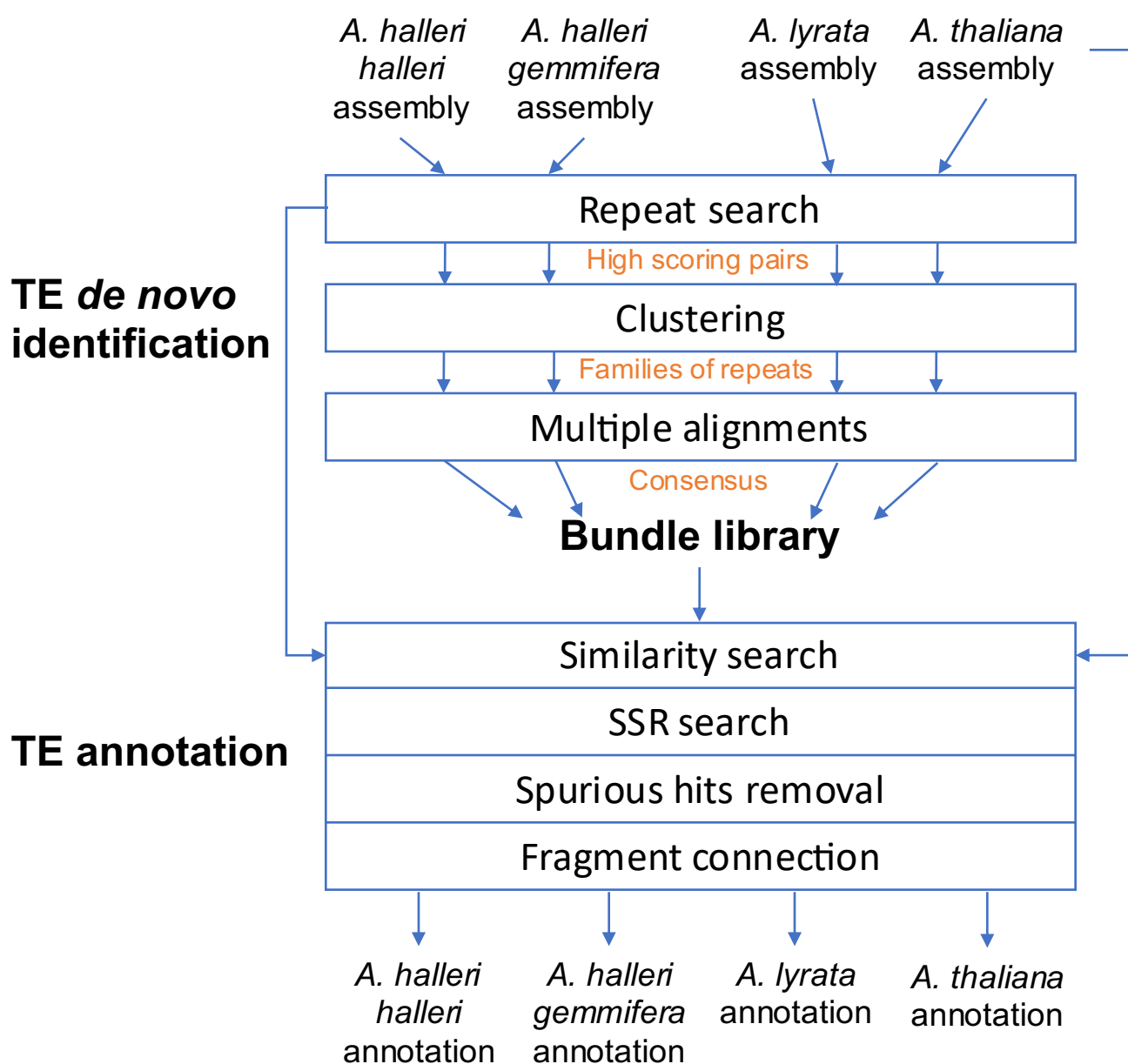

Supplement: Supplementary file 5 — Pipeline used for the deep repeatome annotation. (PDF 30 kb) [file 13100_2019_171_MOESM5_ESM.pdf]

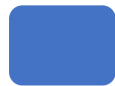

gene

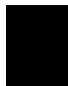

TE

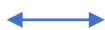

gene orthology  
relationship

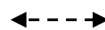

TE orthology  
relationship

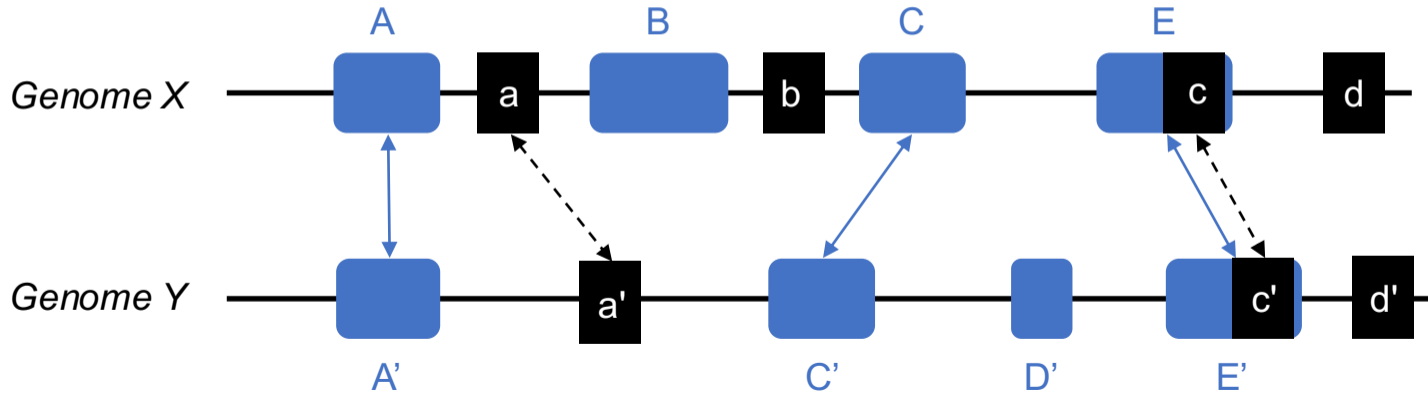

Supplement: Supplementary file 6 — Strategy to identify orthology relationships of TE sequences as determined by positional information from the flanking genes. The first step consists in defining the orthology of genes (blue squares) between genomes X and Y using Inparanoid. In our example, A/A’, C/C′ and E/E’ are considered as orthologous pair of genes (represented by blue arrows). The orthology of TEs is defined sequentially for genome X and Y but the process are similar: only TEs between two orthologous genes spaced for at most 70 kb (black squares named a and b in our example) (named “Framed”) and TEs located within genes (black square c) (named “Inserted”) are analysed. TEs which are located at an extremity of a scaffold (d and d’) and TEs located on scaffold without orthologous genes are discarded. The sequence of the TE a and b, which are located between A and C genes of the orthology map are compared using Blastn (thresholds: Evalue ≤1E− 10, an identity ≥80%) to the sequence between the orthologous genes of A and C, i.e. A’ and C′. The TE a presents a blast hit, and a TE annotation overlaps the Blast hit in genome Y. Hence a and a’ are defined as orthologous. No-significant blast hit is retrieved for b, which is defined as non-orthologous. The sequence of the TE c located within the E gene is compared to the sequence of the E’ gene. In our example, we considered that the Blast hit is significant and overlaps a TE annotation in Genome Y. The TE c is defined as orthologous. (PDF 11 kb) [file 13100_2019_171_MOESM6_ESM.pdf]
